# Multi-omics profiling of CSF from spinal muscular atrophy type 3 patients after nusinersen treatment: a 2-year follow-up multicenter retrospective study

**DOI:** 10.1007/s00018-023-04885-7

**Published:** 2023-08-05

**Authors:** Irene Faravelli, Delia Gagliardi, Elena Abati, Megi Meneri, Jessica Ongaro, Francesca Magri, Valeria Parente, Lucia Petrozzi, Giulia Ricci, Fiorenza Farè, Giulia Garrone, Manuela Fontana, Donatella Caruso, Gabriele Siciliano, Giacomo Pietro Comi, Alessandra Govoni, Stefania Corti, Linda Ottoboni

**Affiliations:** 1 Department of Pathophysiology and Transplantation (DEPT), Dino Ferrari Centre, University of Milan, Milan, Italy; 2 Neurology Unit, Fondazione IRCCS Ca’ Granda Ospedale Maggiore Policlinico, Milan, Italy; 3 Department of Clinical and Experimental Medicine, Neurological Clinics, University of Pisa, Pisa, Italy; 4 Unitech OMICs, University of Milan, Milan, Italy; 5 Department of Pharmacological and Biomolecular Sciences, Università degli Studi di Milano, Milan, Italy

**Keywords:** Spinal muscular atrophy, Antisense oligonucleotides, Proteomic, Metabolomic

## Abstract

Spinal muscular atrophy (SMA) is a neurodegenerative disorder caused by mutations in the *SMN1* gene resulting in reduced levels of the SMN protein. Nusinersen, the first antisense oligonucleotide (ASO) approved for SMA treatment, binds to the *SMN2* gene, paralogue to *SMN1*, and mediates the translation of a functional SMN protein. Here, we used longitudinal high-resolution mass spectrometry (MS) to assess both global proteome and metabolome in cerebrospinal fluid (CSF) from ten SMA type 3 patients, with the aim of identifying novel readouts of pharmacodynamic/response to treatment and predictive markers of treatment response. Patients had a median age of 33.5 [29.5; 38.25] years, and 80% of them were ambulant at time of the enrolment, with a median HFMSE score of 37.5 [25.75; 50.75]. Untargeted CSF proteome and metabolome were measured using high-resolution MS (nLC-HRMS) on CSF samples obtained before treatment (T0) and after 2 years of follow-up (T22). A total of 26 proteins were found to be differentially expressed between T0 and T22 upon VSN normalization and LIMMA differential analysis, accounting for paired replica. Notably, key markers of the insulin-growth factor signaling pathway were upregulated after treatment together with selective modulation of key transcription regulators. Using CombiROC multimarker signature analysis, we suggest that detecting a reduction of SEMA6A and an increase of COL1A2 and GRIA4 might reflect therapeutic efficacy of nusinersen. Longitudinal metabolome profiling, analyzed with paired *t*-Test, showed a significant shift for some aminoacid utilization induced by treatment, whereas other metabolites were largely unchanged. Together, these data suggest perturbation upon nusinersen treatment still sustained after 22 months of follow-up and confirm the utility of CSF multi-omic profiling as pharmacodynamic biomarker for SMA type 3. Nonetheless, validation studies are needed to confirm this evidence in a larger sample size and to further dissect combined markers of response to treatment.

**Supplementary Information:**

The online version contains supplementary material available at 10.1007/s00018-023-04885-7.

## Introduction

Spinal muscular atrophy (SMA) is an inherited neurodegenerative disease and the leading genetic cause of pediatric mortality [1, 2]. The causative mutations of SMA occur in the *Survival Motor Neuron-1 (SMN1)* gene encoding for the protein SMN, which exerts a key role in motor neuron development, function, and survival [3, 4]. Nonetheless, SMA phenotypic spectrum is not restricted to motor neuron-related symptoms [5, 6], and recent evidence suggests a broader systemic involvement, encompassing glial cells and other organs such as heart, kidney, liver, pancreas, spleen, bone, connective tissues, and the immune system [6]. Disease severity may vary greatly among patients, with a phenotypic spectrum that spans from early-life lethal forms (type 1 SMA) to milder phenotypes with longer survival but still significant disability (type 3 SMA). These differences are mainly due to the residual activity of SMN protein, which is related to the number of copies of the *SMN1* paralogue, *SMN2* [7–10].

The peculiar genetic architecture of SMA led to the development of innovative therapeutic strategies [4, 11]. The first disease-modifying therapy for SMA approved by regulatory agencies was nusinersen, an antisense oligonucleotide (ASO) that acts on SM*N2* exon 7 splicing [12] and promotes its retention [13]. Patients with type 1 SMA treated with nusinersen experienced remarkable improvements in the achievement of motor milestones and in overall survival [13] and encouraging results have been provided for adult SMA patients as well [14]. SMA type 3 treated individuals were reported to exhibit a more pronounced improvement on HMFSE score compared to type 2 patients [15]. In patients aged 12 years and older, both HFMSE and RULM amelioration occurred earlier (within 6 months) in SMA type 3 or 4 [16].

To date, several putative biomarkers of disease activity and treatment response have been proposed and investigated, mostly in targeted proteomic studies. Two recent systematic reviews investigated biomarkers of disease progression and nusinersen therapeutic efficacy in SMA type 2 and 3 patients [17, 18]. Targeted studies on disease biomarkers assessed serum *SMN2* mRNA and protein levels and mainly found relative stability throughout the progression [19–22], with the exception of a study that observed a statistically significant increase in *SMN2* transcripts after 12 months of salbutamol treatment [23]. Czech et al. found that *SMN2* transcripts in the blood strongly correlated with *SMN2* copy number in controls, but not in patients, suggesting that other factors likely play a role in *SMN2* expression regulation in SMA individuals [24].

Six prospective studies analyzed specific serum biomarkers of nusinersen response, including creatinine concentration, creatine kinase (CK) activity, neurofilament light chain (NfL) and neurofilament heavy chain (NfH) [25–28], inflammatory biomarkers [29], S100B protein and neuron-specific enolase (NSE) [30]. Elevation of creatinine and reduction of CK levels appeared to correlate with disease severity and muscle damage [31]. In another study, pediatric and adult SMA patients presented an inflammatory signature in serum that was downregulated after nusinersen treatment [29].

As regards CSF biomarkers, changes in NfL and phosphorylated NfH (pNfH) were observed in some studies, but no correlation with muscle function was found [27, 32–35]. Studies on amyloid β40 (Αβ40) and β42 (Αβ42) levels [30, 36], on NSE [27, 30] and on tau protein [27, 30, 37] yielded inconsistent results. De Wel et al. assessed neuroinflammatory biomarkers—chitotriosidase-1 (CHIT1) and chitinase-3-like protein 1 (YKL-40) in the CSF of SMA type 2 and 3 patients over 22 months of nusinersen treatment, and found that YKL-40 correlated with clinical improvements [35].

Four reports of untargeted proteomic analysis of blood and CSF from SMA patients are available, and we thoroughly reviewed them in [38]. Finkel et al. performed a cross-sectional study on blood samples from a cohort of 108 SMA patients and reported a total of 200 candidate biomarkers that correlated with clinical scores [39]. Three more studies assessed CSF proteomic profiles at baseline and after nusinersen treatment. Kessler et al. used mass spectrometry to assess a cohort of 10 SMA type 2 and 3 patients and reported no significant effect of nusinersen on CSF proteome composition; however, they observed intra-individual differences in the modulation of some proteins related to neuronal or muscular function in responders versus (vs) non-responders [40]. Bianchi et al., identified a significant upregulation of apolipoprotein A1 (APOA1), apolipoprotein E (APOE) and transthyretin (TTR) levels in patients after six months of nusinersen treatment [41]. Schorling and colleagues found that Cathepsin D (CTSD), a lysosomal protease involved in protein degradation in skeletal and heart muscles, was upregulated at baseline in SMA patients compared to controls, and decreased more markedly after nusinersen treatment in the responder group compared to non-responders [42].

As regards metabolomics, two studies were performed using nuclear magnetic resonance (NMR) spectroscopy. Deutsch and colleagues analyzed the effect of nusinersen on urine, serum and CSF metabolomes in a cohort of pediatric SMA patients compared with matched healthy individuals but found no effect of treatment on metabolite composition [43]. Notably, urinary creatinine level was the best indicator for discriminating between SMA patients and healthy subjects. Errico et al. identified metabolic patterns associated with treatment that differed in the pathways involved in energy homeostasis according to disease severity in SMA pediatric patients [44].

Overall, results from available omics studies have provided valuable insights, highlighting the potential effect of nusinersen on glucose/insulin, lipid and energy metabolism, immune modulation and muscle remodeling pathways. However, the results are not consistent among different studies. In addition, there are profound differences in the used methodologies, cohorts, and biologic samples. Hence, the need for deep untargeted matched proteomic and metabolomic analyses remains.

Among the potential biofluids, CSF is a valuable bioanalyte, as it is in direct contact with the target tissue and can be obtained while performing nusinersen administration, without additional invasive procedures. In the present study, we used high-resolution mass spectrometry (MS) to assess a combination of non-targeted state-of-the-art CSF proteome and metabolome profiles of patients with SMA type 3 before and after 22 months of nusinersen treatment, with the aim of identifying novel biomarkers of nusinersen efficacy.

## Results

### Motor performances do not change significantly over the 2-year treatment course

**Fig. 1:**
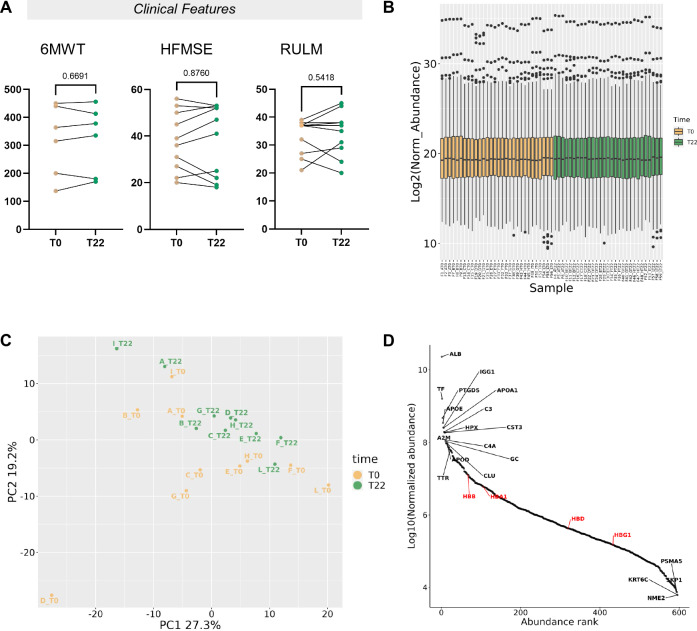
Clinical and CSF proteomic characterization of SMA patients. **A** Changes in HFMSE, RULM and 6MWT scores after the first 22 months of treatment with Nusinersen are reported. Absolute values (higher indicates better) between baseline (yellow) and 22nd month (green) are depicted for each individual patient. **B** Box plots of Log2 Expression of VSN normalized proteomic data, before (yellow, T0) and after treatment (green, T22) are presented. The ten patients are labeled with letters from A to L. Three technical replicates are reported for each sample except for sample I at T22, when only two replicates were available. The VSN normalization produces consistent distributions across all samples and across all replicates, showing no obvious bias. **C** Principal component analysis (PCA) representing unsupervised proteomic data comparative analysis of samples at baseline (yellow label) and 22 months after treatment is plotted. The plot displays a significant separation of the two clusters. Each dot represents the mean average of normalized value of three technical replica for one subject (except for I-T22, in duplicate). Patients are identified with letters from A to L. PCA only based on the top selected features provide a modest separation. **D** Distribution profile for average CSF Log10 normalized abundance of each protein (y-axis) against rank order (from highest that is in position 1, to lowest that is in position 597, x-axis) is shown. Most and less abundant proteins along with hemoglobins (in red) are labeled to represent the dynamic range in the protein mixture

A total of 10 patients with genetic and clinical diagnosis of SMA type 3 were enrolled in this study from 2018 to 2020. Baseline (T0) patient characteristics are reported in Table 1; patients had a median age of 33.5 [29.5; 38.25] years and 80% of them were ambulant at time of the enrolment, with a median HFMSE score of 37.5 [25.75; 50.75]. The median age of disease onset was 9.00 [2.75; 15.25] years, and the median age of treatment was 30.5 [25.50; 35.00] years. Nusinersen was administered every 4 months through lumbar puncture, following the four initial loading doses, and clinical assessment was performed after each treatment visit. Consistent with previous studies, we observed heterogeneous improvements in motor function after 22 months (T22) from treatment initiation, with increased scores in the H FMSE in 5 (50.0%; median of efficacy: 1.0 [– 4.25; 5.50]) patients, in the RULM in 5 (50.0%; median of efficacy: 0.50 [– 2.75; 6.25]) patients and in the 6MWT in 4 (66.7%; median of efficacy: 10.00 [– 21.75; 23.25]) tested patients (Fig. 1A). We defined as responders all patients with an improvement of at least 3 points in HFMSE, as previously suggested by [45]. In supplementary analyses, we also evaluated the effects of nusinersen on RULM; in such analyses, we considered as responders patients with at least one improvement point in RULM scores at T22. In a similar manner, we conducted explorative analyses on the 6MWT parameter: given the lack of consensus on 6MWT among ambulant SMA patients [46], we considered as responders those with increased walking distance at T22.

**Table 1:** Baseline clinical parameters

Clinical features	SMA III (*n* = 10)
Female *S*ex—*n*° (%)	2 (20%)
Age at onset—years	9 [2.75; 15.25]
Age at treatment—years	30.5 [25.5; 35.0]
Disease duration—years	19.5 [17.3; 26.8]
*SMN2 Copy Num—n° (%)*	
2	1 (10%)
3	5 (50%)
4	4 (40%)
Ambulant—*n*° (%)	8 (80%)
Respiratory involv.—*n*° (%)	2 (20%)
HFMSE (score)	37.5 [25.75; 50.75]
RULM (score)	37 [26.50; 37.25]
6MWT (m)	339.5 [184.3; 442.5]

Absolute value (percentage); median [IQR]

### Proteomic profiling of CSF reveals distinct clusters of proteins remodeled by treatment

On average, untargeted MS proteomic analyses allowed the identification and relative quantification of 597 proteins across all CSF samples (Suppl. Table 1 and Suppl. Fig. 1). Normalized abundance is reported in Fig. 1B; the normalization process aims to eliminate any potential skewness among samples, while preserving the biological variability. The diagnostic box plots illustrate the distribution of protein expression in each sample and allow the identification of possible outliers. All samples presented, upon normalization, a very similar distribution. The Principal component analysis (PCA) of all samples, another type of exploratory diagnostic plot, displays the differences of protein expression between the two sample groups, T0 and T22, with a moderate separation after treatment in the first two principal components (PCs; Fig. 1C). Indeed, when accounting for the expression of all hundreds of proteins in each sample, the reduction of the complexity of the system to two summary major principal variables explained less than 50% of the variability of the system (46.5%). The normalized protein intensities spanned over six orders of magnitude, from albumin, the most abundant ranking in position 1, to NME2 (nucleoside di-phosphate kinase B), the least abundant ranking in position 597; the top ten most abundant proteins (1.7% of 597 identified, 596 annotated proteins) contributed to 5% of total protein intensity, the top 60 (10%) to 13.6%, and the top 149 (25%) to 30.8% (Fig. 1D). This distribution pattern is in line with the characteristics of CSF specimens. Albumin was the most abundant protein, followed by Transferrin, Prostaglandin D2 synthase, Apolipoprotein E, Apolipoprotein A1 (ApoA-1), Complement C3, Hemopexin, Cystatin C, and Complement 4A.

**Fig. 2:**
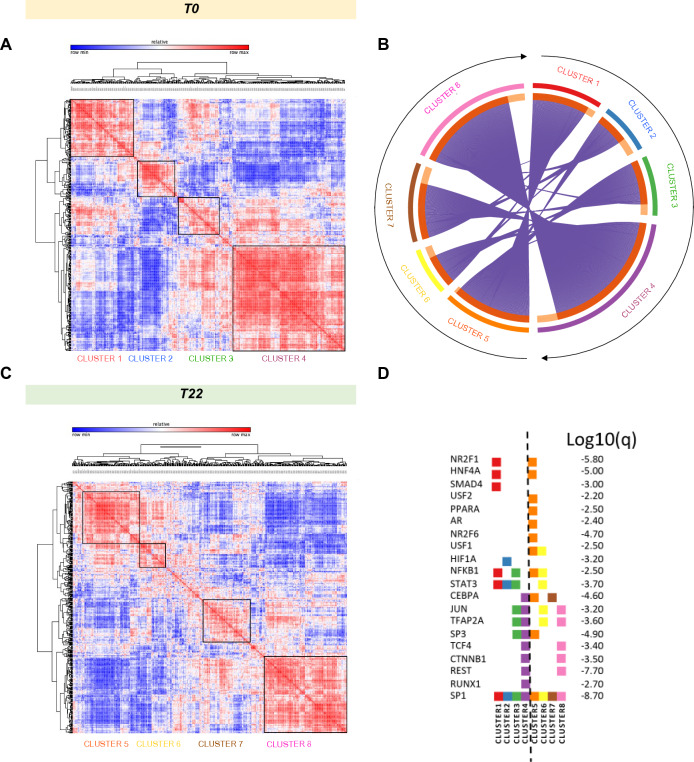
Cross-sectional CSF proteomic analysis. **A**, **C** Correlation maps of all proteins generated by clustering with Euclidean distance the Pearson correlation coefficients of all possible protein combinations at baseline and at T22, after nusinersen treatment are reported. On x- and y-axis all shared proteins are reported in the same sequential order after clustering to highlight the stronger correlation of expression for each time point. The abundance of proteins with similar regulation correlates across samples and forms clusters. Red corresponds to strong correlation and blue to divergent expression. Prominent clusters of proteins with high correlation are framed with a black square. Each cluster has been identified with a numeric color-coded label, cluster 1–4 for T0 and 5–8 for T22. **B** The circos plot (Metascape) shows how proteins from each cluster overlap. On the outside, each arc represents the identity of each protein list, using the same color code as the color used for the cluster identified in (**A**, **C**). On the inside, each arc represents a protein list, where each protein member of that list is assigned a spot on the arc. Dark orange color is for proteins that are shared by multiple lists and light orange color for proteins that are unique to that list. Purple lines link the same protein in different clusters specific for each time point(T0 and T22). The greater the number of purple links and the longer the dark orange arcs imply greater overlap among the input protein lists within cluster. **D** Protein lists are used to perform enrichments in the TRRUST ontology source as implemented in Metascape. Proteins are considered as genes. Q-values of enrichment calculated using the Benjamini–Hochberg correction to account for multiple testing are reported for 20 out of 24 identified significant enriched TRRUST GO (*p* < 0.01). Transcription factors regulating a subset of proteins in each cluster are listed on the left and ordered based on similarity of *p*-value enrichment (full data results in Suppl. Table 4). The TF SP1 is common to all clusters, while HIF1A is enriched exclusively in one cluster, and is therefore likely a signaling pathway specifically active a T0

To gain functionally insights into the co-regulation of proteins in each condition, we generated correlation maps by clustering the Pearson correlation coefficients of all possible protein combinations at T0 and at T22, after nusinersen treatment (Fig. 2). We found that after treatment proteins clustered differently (Fig. 2A, C), we could identify four major clusters in each correlation matrix. Notably, some proteins moved from one cluster to another between T0 and T22 (Fig. 2B). Cluster 4 (violet), the largest at T0, includes proteins that enrich the most for axon development and matrisome as classified by gene ontology (GO) annotation (*q* value < 10^–20^, Suppl. Table 2). Most of these proteins (113 over 194, Suppl. Table 2) are co-correlated also in cluster 8 (pink) at T22 (113 over 147, Suppl. Table 2) and confirm GO enrichment for axon development and axonogenesis pathways (*q* value < 10^–20^, Suppl. Table 2). Similarly, overlap exists between cluster 1 (71 over 108, Suppl. Table 3) and 5 (71 over 121, Suppl. Table 2) where GO enrichment identifies complement-associated pathways (*q* value < 10^–30^ for both clusters, Suppl. Table 2). Co-expressed proteins of cluster 2 and 3 at T0 are mainly re-organized in other clusters or left alone.

We then assessed the most important transcription factors (TFs) regulating proteins in the different clusters (the top 20 are reported in Fig. 2D). Correlated proteins in clusters 4 and 8 shared GO annotations for axon development and similar pathways among the ones identified (Suppl. Table 3), and they are linked to shared TFs, including JUN, TFAP2, TCF4, CTNNB1, REST, and SP1. Interestingly, more transcription factor remodeling could be detected when comparing clusters 1 and 5, which shared only NR2F1, HNF4A, NFKB1, and SP1. SMAD4 was specific for cluster 1, while USF2, PPARA, AR, and NR2F6 were enriched after treatment and only present in cluster 5, suggesting that nusinersen specifically impacts signaling that are controlled by those TFs (Suppl. Table 3). Interestingly, HIF1α, SMAD4, and RUNX1 were quenched after treatment.

**Fig. 3:**
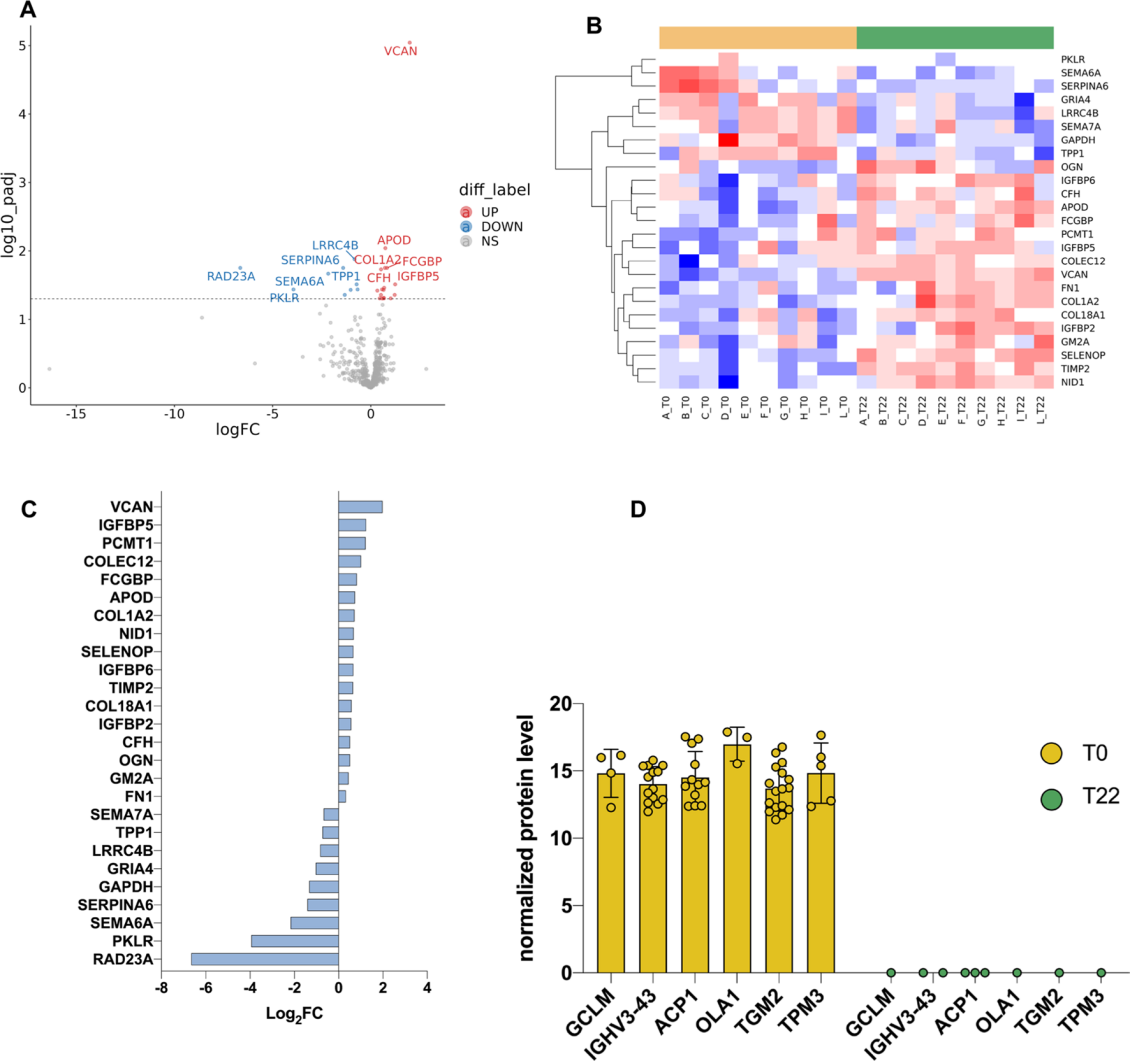
Nusinersen treatment consequences at protein level. **A** Treatment vs Baseline protein fold changes are plotted versus log10 *p*value (y axis) in a volcano plot. Proteins above the dashed line are statistically significantly different (*p*Adj < 0.05), and those depicted in red (up) are more abundant upon treatment (*n* = 17) while those in blue (down) are reduced (*n* = 9) after treatment. On the x axis, the log fold change of expression is represented. **B** Heatmap of hierarchical clustering for significantly differentially expressed proteins (*p*Adj < 0.05) unveils two major clusters of proteins upregulated either at baseline (yellow) or after 22 months of treatment (green). Color code expression levels are scaled by row, with red color corresponding to high expression, blue color to low expression and white to undetected protein. RAD23A protein was not included in the heatmap because it was detected in sample F in all three technical replicates at baseline while only in one sample upon Nusinersen treatment. **C** The bar plot reports the ranked list of fold changes (x-axis) between T22 and baseline of significantly differentially expressed proteins (y-axis). Protein RAD23A is here included. Of note, RAD23A turns out to be the protein with the largest fold change. **D** A set of 6 proteins has been detected at baseline, in at least one subject and in at least one technical MS detection (clear yellow dot). The same proteins completely disappeared at T22 in all subjects (green dot)

CSF analysis resulted in the identification of 26 proteins differentially expressed (DEP) after treatment, depicted as upregulated (red, *n* = 17) or downregulated (blue, *n* = 9) in the volcano plot (Fig. 3A). Hierarchical clustering of DEPs led to the identification of two major clusters (Fig. 3B).

Versican core protein (VCAN), a large extracellular matrix proteoglycan, displayed the highest expression after treatment, while UV excision repair protein RAD23 homolog A (RAD23A) was the most downregulated, although only in one subject. Similarly, pyruvate kinase liver and RBC (PKLR), a glycolytic enzyme that catalyzes the trans-phosphorylation from phosphoenolpyruvate (PEP) to ADP [47], were significantly reduced upon treatment in one subject.

SEMA6A was instead identified among the most reduced targets across all subjects (Fig. 3C). Similarly, SEMA7A, a protein belonging to the same family as SEMA6A, was also observed to be downregulated (Fig. 3C). Interestingly, Kessler et al. reported SEMA7A as upregulated in stable/non responder patients after treatment, and thus downregulated in responders after 10 months of nusinersen treatment [40].

Of note, three insulin-like growth factor binding proteins (IGFBP5, IGFBP6, IGFBP2) were upregulated after treatment, while six proteins (GCLM, IGHV3-43, ACP1, OLA1, TGM2, TPM3) found in CSF at baseline were not detected at all in any of the samples collected at T22 (Fig. 3D).

### Pathway analysis of deregulated proteins

**Fig. 4:**
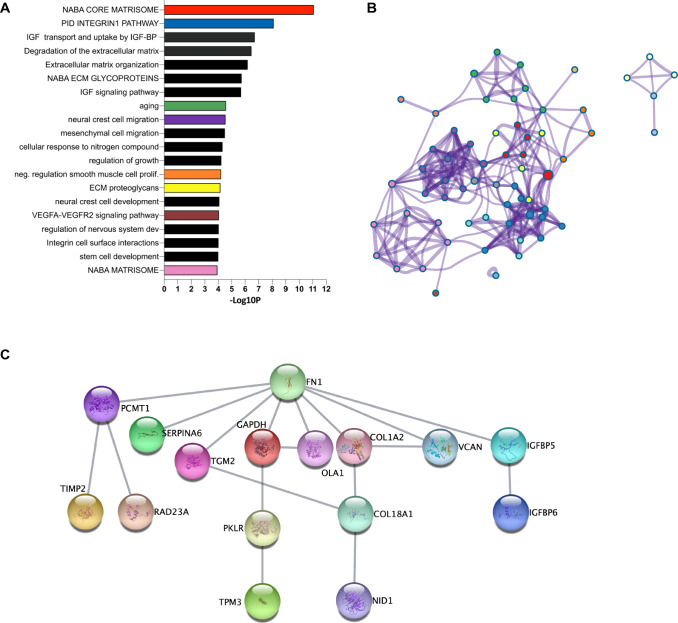
Pathway analysis results of differentially expressed proteins before and after treatment. **A** The bar plot reports the ranked results (– log10 *p* value, x-axis) of the top 20 selected enriched terms (colored bars) among clusters after pathway and process enrichment analysis carried out with the following ontology sources: KEGG Pathway, GO Biological Processes, Reactome Gene Sets, Canonical Pathways, CORUM, WikiPathways and PANTHER Pathway as performed in Metascape using the list of significant differentially expressed proteins and 6 proteins detected only at baseline as described in Fig. 3. **B** A subset of top significant clusters were converted into a network layout, colored by cluster ID, where nodes (pathways) that share the same cluster ID are typically close to each other. Each term of a cluster is represented by a circle node, where its color represents its cluster identity (i.e., nodes of the same color belong to the same cluster) and its size is proportional to the number of input proteins that fall under that term. Terms with a similarity score > 0.3 are linked by an edge (the thickness of the edge represents the similarity score). The network is visualized with Cytoscape with “force-directed” layout and with the edge bundled. **C** All protein–protein physical interactions among input proteins (26 + 6) were extracted from PPI data source and formed a PPI network using STRING and BioGrid. The hierarchical representation is generated in Cytoscape

Next, we sought to understand whether modulation of significantly DEPs upon treatment might be linked to specific signaling pathways to discover downstream cascades modified by the treatment. Through mature complex identification algorithm (MCODE) analysis, we were able to extract putative functional biological roles for the deregulated proteins (Fig. 4A, Suppl. Table 4). Most of the identified pathways (*q* value < 0.05) were related to extracellular matrix composition, but also cellular migration and central nervous system (CNS) development. Interestingly, we found that insulin-growth factor (IGF) signaling and metabolism were among the enriched pathways after treatment, with three IGFBPs supporting this enrichment. Network representation of interacting clusters is reported in Fig. 4B, where color-coding correspondence to the ranked bar plot is depicted. Of note, DEP proteins were involved in several interesting contexts, such as integrin activation, neural crest migration, smooth muscle proliferation, generally linked to the dynamic microenvironment that influences cellular functions and tissue development.

Protein–protein direct connections were interrogated with STRING, which reports the subset of proteins that form physical interactions with at least one other member of the dataset. Fibronectin (FN1) showed the highest degree of connection (Fig. 4C).

### Clinical and proteomic profiling correlation

**Fig. 5:**
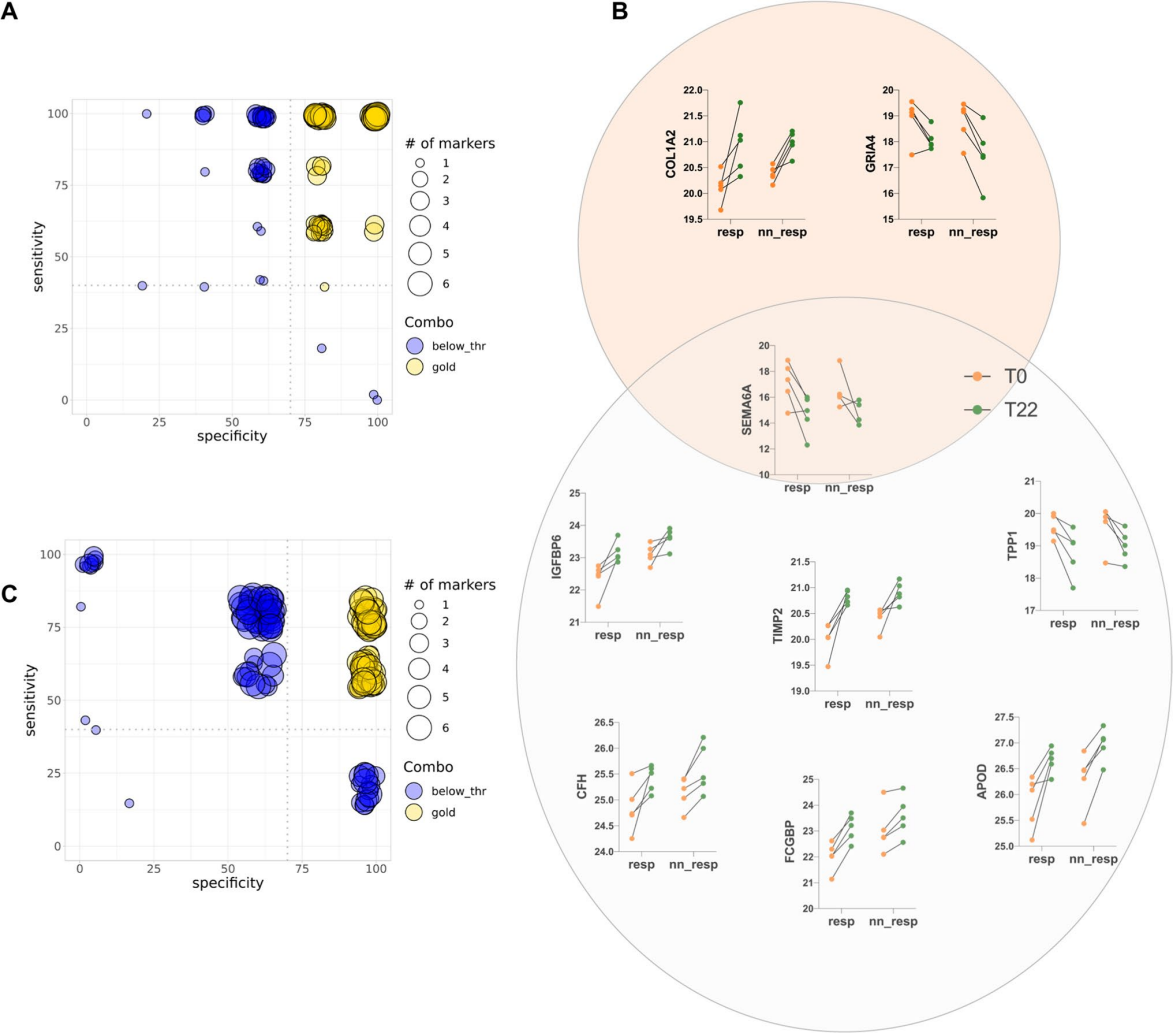
Combinatorial selection of differentially expressed proteins as biomarkers to optimize protein signatures in diagnostics applications. **A** Bubble plot of CombiROC analysis performed with combination of up to 6 proteins leveraging differential values of 26 significant differential proteins and classification of responder and non-responder according to HFMSE clinical score. Blue circles correspond to combinations with specificity < 70% and sensitivity < 40%, while yellow circles (“Gold”) to specificity > 70% and sensitivity > 40%. **B** Shared biomarkers among optimal Combiroc combinations with specificity > 70% and sensitivity > 40% and combination of 3 markers as in (**A**). Dot plots for selected proteins are reported for responders and non-responder at baseline (orange) and after treatment (green). **C** Bubble plot of CombiROC analysis performed with combination of up to 6 proteins leveraging values of 26 significant differential proteins at T0 and classification of responder and non-responder according to HFMSE clinical score. Blue circles correspond to combinations with specificity < 70% and sensitivity < 40%, while yellow circles to specificity > 70% and sensitivity > 40%. **D** Shared biomarkers among optimal Combiroc combinations with specificity > 70% and sensitivity > 40% and combination of 3 markers as in (**C**). Dot plots for selected proteins are reported for responders and non-responder at baseline (orange) and after treatment (green). SEMA6A is shared markers in both analyses, leveraging differential change as well as predictive value at T0

Next, we investigated whether changes in specific proteins could be correlated with the clinical outcome. We analyzed the changes in CSF proteomics within each individual and investigated their association with treatment response based on clinical assessments. For this purpose, we created a matrix that contained the differential values for DEP after nusinersen treatment compared to before treatment (T22-T0) for each patient. The CombiROC analysis, which tested for responder *vs* non-responders relative to HFMSE, identified a combination of up to 6 protein markers as predictors of clinical response (Fig. 5A).

Considering the combination with 100% specificity and 100% sensitivity as the best solution, 93 combinations were found: 1 with only 3 markers, 8 with 4 markers, 28 with 5 markers, and 56 with 6 markers (Suppl. Table 5). Considering the combinations with at least 3 markers, which might still be suitable in clinical practice, SEMA6A, COL1A2, and GRIA4 were unique and shared. Their trends and the difference before and after treatment in responders and non-responders are reported in Fig. 5B. The approach suggests that detecting a reduction of SEMA6A and an increase of COL1A2 and GRIA4, of a given fold change, might reflect therapeutic efficacy of nusinersen. Of note, nusinersen effect on COL1A2 levels was reported in a previous study on SMA type 3 patients [40].

Intraindividual proteomic changes were also tested for RULM and 6MWT clinical outcomes to identify predictive biomarker combinations (Suppl. Fig. 1). While RULM analysis exploited several significant combinations (Suppl. Fig. 2A), 6MWT allowed only the identification of combinations with at best 80% specificity and 40% sensitivity (Suppl. Fig. 2B).

However, the intraindividual proteomic change approach has limited utility for personalized prediction of the therapeutic efficacy. Thus, we tested CombiROC on T0 proteomic data and in correlation with HFMSE modulation, identifying 1 combination of 2, 6 combinations of 3 and 15 combinations of 4 proteomic markers with at best 100% specificity and 80% sensitivity (Suppl. Table 6). All unique proteomic markers for combinations of 3 are reported in Fig. 5C. Except for TPP1, whose trend after treatment is increasing, for all other markers the trend is toward reduction. Of note SEMA6A is the only shared target when testing prediction using intraindividual proteomic changes or T0 proteomic value. Validation of the magnitude of reduction on a much larger cohort of patients is needed to confirm whether SEMA6A might be a predictive biomarker. Longitudinal assessment at consecutive infusions will also be important.

### Metabolome profiling of CSF reveals distinct clusters of analytes deregulated by the treatment

**Fig. 6:**
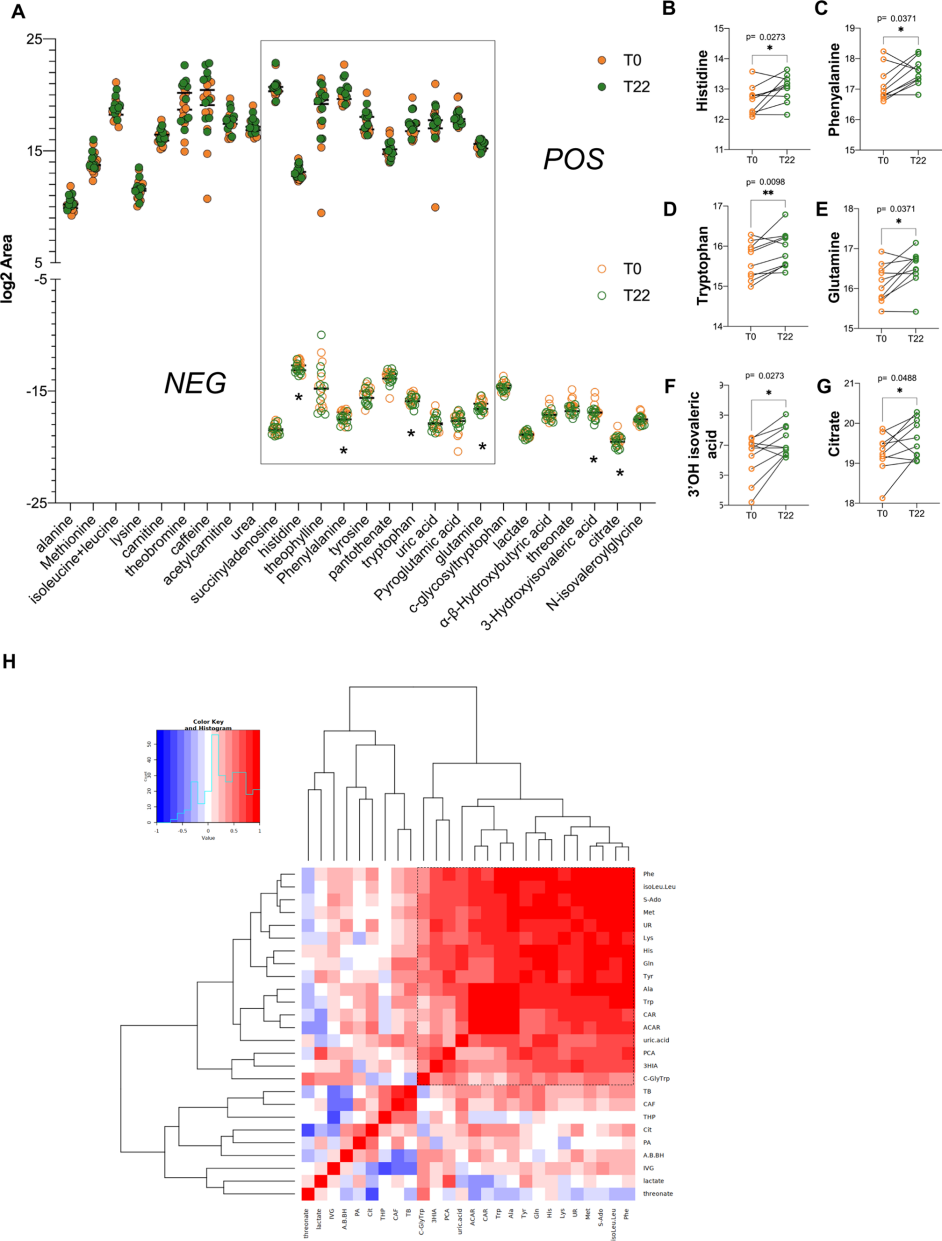
Cross-sectional CSF metabolic analysis. **A** Dot plot distribution of metabolites detected with best confidence, positively and negatively charged. In the grey frame metabolites detected in both modes are highlighted. * = significant change. **B**–**G** Paired dot plot at baseline and after treatment of metabolic features that significantly change after treatment. **H** Pearson correlation matrix of metabolite abundance difference between T22 and T0. Most co-modulated features cluster in the frame with dashed lines

Metabolomic analysis of the same CSF specimens from SMA type 3 patients at T0 and T22 identified 45 unique metabolites, 19 with high confidence and 11 with low confidence, positively charged, and 17 with high confidence and 11 with low confidence, negatively charged (Fig. 6A). Out of the metabolites detected with high confidence, 10 were detected as positively or negatively charged (Fig. 6A), while among those with low confidence only one was detected in both modes (Suppl. Fig. 3G). We identified 6 significantly different negatively charged metabolites after treatment compared to baseline. Four of them (histidine *p* = 0.0273, phenylalanine *p* = 0.0371, tryptophan *p* = 0.0098, glutamine *p* = 0.0371) were amino acids whose levels increased after treatment (Fig. 6A–G). Furthermore, 3-hydroxyisovaleric acid (*p* = 0.0273) and citrate (*p* = 0.0488) tended to increase after treatment as well.

While hierarchical clustering did not identify any specific correlation among metabolite levels (Suppl. Fig. 3), we were able to identify a significant cluster of correlations by conducting Pearson correlation analysis of the differences in metabolite levels (T22-T0). This cluster was enriched for pathways associated with aminoacyl–tRNA biosynthesis (FDR 6.85 × 10^–12^) and phenylalanine, tyrosine and tryptophan biosynthesis (FDR 0.016) (Fig. 6H and Suppl. Table 7).

Additionally, among metabolites detected with lower confidence, positively charged stearoyl sphingomyelin was significantly reduced, a result that might be in line with SEMA6A reduction in the context of myelination (Suppl. Fig. 4G, H).

When accounting for clinical profile based on HFMSE score, we did not detect any significant change after treatment in responders compared to non-responders (Suppl. Fig. A–F). Overall, these findings point toward a metabolic effect of the treatment with nusinersen over an extended period of time with a major impact on amino acids.

## Discussion

In this study, we explored the biochemical profile of treated SMA patients by combining mass spectrometry-based proteomics, metabolomics, and bioinformatic analyses. Our analysis identified 26 differentially expressed proteins in the CSF between T0 and T22. We propose that detecting a reduction in SEMA6A, along with an increase in COL1A2 and GRIA4, may serve as indicators of therapeutic efficacy of nusinersen. Furthermore, longitudinal metabolome profiling revealed significant changes in amino acid utilization induced by the treatment.

Therapeutic trials conducted in pediatric patients with SMA showcased a significant benefit on motor functions in patients treated with nusinersen compared with sham procedure [13, 40, 48]. Notwithstanding, it is still a matter of debate whether adult SMA patients with slowly progressing disease may also improve, with a meaningful significant magnitude, following nusinersen therapy.

Existing literature showcases varying results, with most studies reporting a sustained improvement in motor scales after 24 months of follow-up [14, 30, 49, 50], and a few others observing only a stabilization in motor function, with prevention of further decline.

Available clinical and omics studies on SMA population are mostly targeted approaches, and only a few non-targeted proteomic studies have been published so far [38]. In addition, the studies exploring metabolomic readouts [43, 44] were performed with NMR, which provides reduced sensitivity in the identification of metabolites present at nM concentrations. Overall, these results suggest a potential effect of nusinersen on glucose/insulin, lipid and energy metabolism, immune modulation, and muscle remodeling pathways. These few studies demonstrate the feasibility of an omics approach, but they carry some limitations, such as a short follow-up period, a low number of patients, and assessment of samples at only two time points. This could partially explain why there is very little overlap of metabolites and proteins among studies, suggesting varied proteo- and metabolic-omic modulation early on after treatment. The use of multi-omic bioinformatic analysis, which integrates metabolomic and proteomic techniques, could strengthen the validity of these findings, providing meaningful information.

In our cohort, we used a comprehensive MS-based study assessing in a non-targeted fashion both proteome and metabolome on CSF of patients with SMA type 3. We found modifications in the proteome and metabolome of SMA patients after 22 months of treatment with nusinersen compared with the baseline. DEPs clustered differently according to the time point, with remodeling of the four main co-correlated groups of proteins. Notably, these clusters showed expression differences in pathways related to inflammation (JUN, NFKβ, HIF1α), suggesting a treatment-specific effect on these molecular cascades. These findings suggest that nusinersen might have a non-motor neuron specific positive effect due to the improvement in inflammatory response.

We observed the presence of differentially expressed proteins according to the time point, with 17 proteins being significantly upregulated and 9 significantly downregulated after treatment. Among proteins whose expression was significantly upregulated after 22 months there are VCAN, which displayed the highest expression, ApoD, SELENOP, and IGFBP5/IGFBP6. VCAN participates in intercellular signaling and is involved in different processes, including cell adhesion, proliferation, migration, and connection to the extracellular matrix and plays a key role in neuronal differentiation and neurite outgrowth [51]. Consistently, the pathways by the MCODE analysis were mainly related with extracellular matrix composition, cellular migration, and neurodevelopment.

Apolipoprotein D (ApoD) is a glycoprotein of the lipocalin family expressed in glial cells of the central and peripheral nervous system and mostly associated with lipid metabolism and neuroprotection [52, 53]. Thus, our findings on ApoD upregulation in the CSF of treated SMA type 3 patients might indicate an initial nusinersen-induced restoration of disrupted lipid metabolism in SMA, which, if prolonged, might lead to the rescue of neuronal processes including neurite outgrowth, neuronal survival and plasticity and, ultimately, to clinical improvement. Moreover, ApoD plays also a pivotal role both in the age-dependent maintenance of peripheral nerve function and tissue homeostasis and in the response to injury, by promoting myelin clearance, modulating angiogenesis and recruitment of inflammatory cells as well as axonal regeneration and remyelination [54]. These results highlight the potential benefits of nusinersen administration in patients with SMA type 3, including those with disease onset occurring after childhood. Additionally, our findings align with previous research conducted by Bianchi et al., that identified a significant upregulation of ApoA1 and ApoE after nusinersen treatment, pointing at a therapeutic effect upon lipid metabolism and inflammatory pathways, and Bonanno et al., that reported a modulation of the inflammatory activation in SMA-treated patients [29, 41]. As regards seleno-protein P, it is mainly a liver expressed protein that acts as an extracellular antioxidant and as selenium transporter to extrahepatic tissues. However, several pieces of evidence suggest that it is also relevant for normal brain function [55]. In addition, low CSF concentrations of selenoprotein P-bound selenium have been associated with increased risk of amyotrophic lateral sclerosis [56, 57], a sporadic adulthood onset motor neuron disorder. Consistently, we found increased CSF concentrations in SMA patients after nusinersen administration.

Of note, members of the innate immune complement pathways, both activators (C3, C4A) and inhibitors (SERPING1), were among the most abundant CSF proteins, triggering several questions on their functions. As nicely reviewed in [58], recent studies looked at the involvement of the complement C1q factor in the degeneration of SMA motor neuron synapses, while its pharmacological inhibition was able to rescue proprioceptive synapses in a mouse model of SMA and ameliorated motor deficits.

As regards instead proteins whose expression in CSF was significantly decreased compared to baseline, SEMA6A, a member of the semaphorin family, essential for axonal guidance, with a central role during spinal cord development, was identified [59–61]. Aberrant motor neuron migration during development has been implicated in SMA pathogenesis due to a lack of proper connection from the corticospinal fibers [62, 63]; these findings correlate with the central role of SEMA6A in axons.

In addition to neurodevelopment, SEMA6A is involved in oligodendrocyte differentiation and myelination [64]. Together with SEMA4D and SEMA6, its expression has been found upregulated in oligodendrocytes close to an injury site [65]. Notably, SEMA7A, a member of the semaphorin family involved in axonal guidance and immunomodulation, was found to be enriched in the CSF proteome of patients who responded better to nusinersen treatment in the study by Kessler and colleagues [40].

Interestingly, a group of proteins was no longer detected after treatment. Among them, OLA1, a novel Obg-like ATPase, functions as a negative regulator of the cellular antioxidant response independent of transcriptional processes [66]. The beneficial effects observed upon OLA1-knockdown suggest that this regulatory ATPase is a potential novel target for antioxidative therapy.

Through MCODE analysis, we identified pathways connected with the differentially expressed proteins. These pathways, which are mainly related to extracellular matrix composition, cellular migration and neurodevelopment, include the IGF signaling pathway, whose overexpression was previously shown to improve disease features in SMA preclinical models [67, 68].

When assessing the correlation between differentially expressed proteins and clinical outcome measured with HFMSE, we were able to appraise that the SEMA6A CSF reduction from T0 to T22 might be a predictor of clinical outcome.

The metabolome analyses revealed a modification in the aminoacidic profile upon nusinersen treatment. 3-Hydroxyisovaleric acid is a by-product of the leucine degradation pathway, which is important for glutamate homeostasis. Of note, both 3OH isovaleric acid and glutamine increase after treatment. Thus, the observed increased levels of isovaleric acid at T22 might suggest increased leucine degradation in favor of glutamine and glutamate production [69, 70]. On the same note, citrate is specifically synthesized by and released from astrocytes and one of its interesting features is its pivotal role as an endogenous modulator of glutamate receptors, in particular the NMDA subtypes of these receptors in the CNS, via Ca^2+^, Mg^2+^ and Zn^2+^ chelation, which impacts the excitable state of neurons [70]. Our findings are in line with a previous metabolomic study by Errico et al., that highlighted the impact of nusinersen on amino acid metabolism in SMA type 3 patients [44].

In summary, MS proteomic/metabolomic approaches uncovered neuroproteomic and metabolic signatures of SMA. Limitations of our study include the small sample size, the evaluation limited to the CSF, and the assessment of the analytes at only two time-points. However, the extended longitudinal follow-up (22 months) enabled the identification of a significant difference in the CSF proteome and metabolome after treatment. Our data suggest a CSF perturbation due to nusinersen treatment still sustained after 22 months of follow-up. Nonetheless, validation studies are needed to confirm this evidence in a larger sample size, ideally with measurement at different time points, potentially extending the analyses to other biological fluids, and to further dissect combined markers of response to treatment.

By linking genotype, proteo-type, metabolism, and phenotype, integrated proteomic and metabolomic approaches provide a new opportunity to improve precision treatment strategies.

## Methods

### Neurological assessment

The Neurology Unit of Fondazione IRCCS Ca’ Granda Ospedale Maggiore Policlinico di Milano and the Neurology Unit of Azienda Ospedaliero Universitaria of Pisa recruited patients with both clinical and genetic diagnosis of SMA type 3. All patients from our cohort have a disease onset after 18 months of age and all but one has 4 or 3 copies of *SMN2*. The patient with 2 copies of *SMN2* holds the missense variant c.389A > G (p.Y130C) in *SMN1*. All patients were monitored throughout a 22-month follow-up, starting before the first nusinersen treatment and on the following administration days (according to the national Agenzia Italiana del Farmaco—AIFA guidelines). All patients were routinely tested with standardized motor function scales: Hammersmith Functional Motor Scale Expanded (HFMSE), 6 Minute Walking Test (6MWT) for ambulant patients and the Revised Upper Limb Module (RULM). Baseline clinical features and efficacy measures were analyzed through descriptive statistics, continuous variables were reported as median [IQR], as appropriate.

The study was conducted in accordance with the Declaration of Helsinki and followed ICH GCP guidelines. All subjects and the parents of minors provided written informed consent for the collection, storage and analysis of biological materials according to the disposition of the local ethics committees.

### CSF mass spectrometry analysis

CSF samples for analysis were collected right before the first nusinersen intrathecal administration (pre-treatment, T0) and on a follow-up administration at 22 months (T22). CSF was collected for all time points and from all patients in the morning, after an overnight fasting. CSF collected after lumbar puncture was aliquoted, frozen within 3 h from collection, and stored at − 80 °C until analysis. All samples have been analyzed at UNITECH OMICs (University of Milano, Italy). Protein concentration of each sample has been quantified using BCA assay. 10 mg of proteins (in Ammonium Bicarbonate 50 mM) for each sample have been first reduced in 5 mM dithiothreitol (DTT) at 55 °C for 30 min, alkylated in 15 mM iodoacetamide (IAA) at R.T. for 20 min and digested with trypsin 0.1 μg/μl at 37 °C overnight with final reaction blocking in formic acid (FA). 5 μg of the protein lysate have been purified with a zip-tip C18 column. The eluted material has been concentrated in a speedvac and reconstituted in 20 μl with FA 0.1%. 4 μl of the reconstituted material have been injected in nLC-HRMS. Each sample has been tested three times except for T22 sample I, which was tested only twice. Samples have been analyzed on Dionex Ultimate 3000 nano-LC system (Sunnyvale CA, USA) connected to Orbitrap Fusion™ Tribrid™ Mass Spectrometer (Thermo Scientific, Bremen, Germany) equipped with nano-electrospray ion source (UNITECH OMICs, University of Milano, Italy). Peptide mixtures were pre-concentrated onto a Acclaim PepMap 100 – 100 μm × 2 cm C18 (Thermo Scientific) and separated on EASY-Spray column ES802A, 25 cm × 75 μm ID packed with Thermo Scientific Acclaim PepMap RSLC C18, 3 μm, 100 Å, at 35 °C. The peptides were eluted with the following gradient: from 96% buffer A (0.1% formic acid in water) to 95% buffer B (0.1% formic acid in water/acetonitrile 2:8) in 110 min at the constant flow rate of 300 nL min − 1 for a total run of 144 min. One blank was run between samples to prevent carryover. MS spectra were collected over an m/z range of 375–1500 Da at 120,000 resolutions, operating in the data dependent mode, cycle time 3 s between master scans. Higher energy C-trap dissociation (HCD) was performed with collision energy set at 35 eV. Polarity mode: positive.

After MS recording, the spectra have been elaborated with Proteome Discoverer 2.4 searching in the uniprot-human-reviewed_1422020.fasta database, applying trypsin enzyme analysis and the following filtering criteria: peptide ≥ 2, Xcorr for peptide ≥ 2.2, Xcorr for PSM ≥ 2.2.

### Metabolomic analysis

Internal standards (Palmitic Acid, Linolenic Acid, Phosphatidylcholine) and 500 µL of extractive phase (methanol:ethanol,1:1 v/v) were added to 100 µL of CSF from each sample. The samples were then kept at – 20 °C for one hour. Subsequently, the samples were centrifuged and 500 µL of supernatant were dried out under nitrogen flow. The extracts were resuspended with 50 µL of methanol and analyzed in UHPLC/MS–MS.

All samples have been analyzed using ExionLC™ AD system (SCIEX) connected to TripleTOF™ 6600 System (SCIEX) equipped with Turbo V™ Ion Source with ESI Probe. Chromatographic separation was achieved on a CORTECS UPLC T3 column (Waters), 150 (Length) × 2.1 mm (ID) × 1.6 µm (Particle Size) using mobile phase A (0.1% formic acid in water) and mobile phase B (0.1% formic acid in acetonitrile) at a flow rate of 400 µL/min in 20 min. The column and autosampler temperatures were set at 40 °C and 7 °C, respectively. The sample injection volume was 5 µL. Each sample was injected two times. MS spectra were collected over an m/z range of 50–1500 Da in positive/negative polarity, operating in IDA®mode (Information Dependent Acquisition). Collision energy was set at 30 (CES 15).

Raw data files (.wiff) were converted into.abf format and processed with MSDIAL Software ver. 4.24 by setting the MSMS-Public-Pos Database (version 15) for analyses conducted in ESI Pos and MSMS-Public-Neg (version 15) for analyses conducted in ESI neg. After background subtraction using a Blank sample, 1572 IDs in positive polarity and 700 ID in negative polarity have been detected. Using a pool QC sample as reference, only IDs with a CV% of mean response area less than 30% were considered as identified in replicates of run (*n* = 10) to obtain 1380 IDs in positive (known and unknown) and 385 IDs in negative (known and unknown). From the total IDs, only those that had an identified MS/MS spectrum were considered, resulting in 202 positive IDs (MS/MS) and 316 negative IDs (MS/MS). IDs have been classified in level of confidence for detection (1–4). IDs scored with 1 or 2 were considered detected with high confidence, IDs with score 3 or 4 were considered detected with low confidence. Analysis of high and low confidence IDs has been performed separately.

### NormalizerDE and differential analysis

NormalizerDE [71] is a software that helps to select an optimal normalization approach to perform subsequent differential expression on LC–MS-based expression data. It includes several normalization approaches, calculates performance measures, and generates an evaluation report. Version 1.5.4 was used. Since in a comprehensive review of different normalization techniques the variance stabilizing normalization (VSN) generally performed best, particularly for metabolomics data [72, 73], the VSN normalization was chosen (Suppl. Table 8). Quality assessment was performed by inspection of boxplots and relative log expression plots. Following these steps, none of the samples was excluded. Singular value decomposition was used to perform principal component analysis (PCA) on the normalized proteomic data using the built in R function. The empirical Bayes-based statistical approach implemented as part of Limma (R Bioconductor) has increased sensitivity over ANOVA in differential analysis [74] and provides corrected pvalue BH (Benjaimin Hockberg) < 0.05. VSN normalized matrix was tested with Limma for differential analysis accounting for paired replica data (R version 4.1.2; Suppl. Table 9).

### Functional enrichment analysis and Interactome analysis

Pearson’s correlation coefficient was calculated in R version 4.1.2 using normalized data matrix T0 and T22 for all possible protein combinations and protein correlation matrixes were represented in hierarchical clustering using GENE-E [75] or R.

General expression analysis was performed in Metascape [76] using the lists of genes corresponding to the major clusters identified in the correlation matrix and the list of significant differentially expressed proteins. Overlaps between lists of genes in clusters are shown in a circos plots [77] implemented using Cytoscape [78]. Further, for each gene list, enrichments are identified in the ontology category TRRUST (transcriptional regulatory relationships unraveled by sentence-based text-mining) [79]. All genes in the genome have been used as the enrichment background. Terms with a *p*-value < 0.01, a minimum count of 3, and an enrichment factor > 1.5 (the enrichment factor is the ratio between the observed counts and the counts expected by chance) are collected and grouped into clusters based on their membership similarities. More specifically, p-values are calculated based on the cumulative hypergeometric distribution and q-values are calculated using the Benjamini–Hochberg procedure to account for multiple testings. The most statistically significant term within a cluster is chosen to represent the cluster. Colored pattern shows the color code used for the gene lists where the term is found statistically significant, i.e., multiple colors indicate a TF that is shared across multiple lists.

As regards the enrichment network analysis on significant differential proteins, to further capture the relationships between the terms, a subset of enriched terms has been selected and rendered as a network plot, where terms with a similarity > 0.3 are connected by edges. The terms with the best p-values from each of the 20 clusters are represented, with the constraint that they are no more than 15 terms per cluster and no more than 250 terms in total. The network is visualized using Cytoscape, where each node represents an enriched term and is colored first by its cluster ID. The resultant network contains the subset of proteins that form physical interactions with at least one other member in the list. If the network contains between 3 and 500 proteins, the molecular complex detection (MCODE) algorithm has been applied to identify densely connected network components [80].

Further, protein–protein interaction analysis has been carried out on DEP (26 and 6 only at T0) using only physical interactions in STRING (physical score > 0.132) and BioGrid. All protein–protein interactions were reported with “force-directed” layout and with edge bundled, reproduced in Cytoscape.

### CombiROC analysis

CombiROC is a tool implemented to accurately determine optimal combinations of markers from complex omics data [81] to classify samples as positive or negative according to a dataset-specific threshold (thres) above which the features’ values are considered positive (the “test’s signal cutoff”). Minf represents the minimum number of features that need to reach the thres. All possible combinations, both as single marker and combination markers, are generated.

For each of the combinations satisfying the thresholds, the sensitivity (SE) and specificity (SP) filters, interpreted in terms of recognition frequency, are computed. SE is defined as the true positive rate in percentage of the sample whereas SP is defined as the true negative rate in control class in percentage. Analysis was performed using differential values between T22 and T0. The best SP (> 70%)/SE (> 40%) values were chosen as “Gold Combinations.”

## Supplementary Information

Below is the link to the electronic supplementary material.

Supplementary Figure 1. Box plot of non-normalized proteomic data, before (yellow, T0) and after treatment (green, T22). Each box plot corresponds to a sample technical replica and describes the distribution of the expression of all proteins detected in each sample. Supplementary Figure 2. A-B. Bubble plots of CombiROC analysis performed testing a combination of up to 6 protein markers among 26 significantly differentially expressed (T22-T0) values in correlation with the classification of responder and non-responder according to RULM and 6MWT clinical score respectively. Blue circles correspond to combinations with specificity <70% and sensitivity <40%, while yellow circles to specificity >70% and sensitivity >40%. Supplementary Figure 3. Hierarchical clustering of normalized detected positive (left panel) and negative (right panel) metabolite level, before (orange bar) and after (green bar) treatment. Supplementary Figure 4. A-F. Paired dot plots for normalized detected positive and negative metabolite levels, before (orange) and after (green) treatment, accounting for HFMSE score (responder and non-responder). G. Dot plot distribution of positive (filled dot) and negative (open dot) metabolites detected with lower confidence, before (orange) and after (green) treatment. In the grey frame metabolites detected in both modes are highlighted. H-I. Paired dot plot for stearoyl sphingomyelin before (orange) and after (green) treatment and accounting for HFMSE score (responder and non-responder)

Supplementary Table 1. Proteins identified in the CSF

Supplementary Table 2. GO enrichment results by cluster from Metascape

Supplementary Table 3. GO TRRUST results from Metascape

Supplementary Table 4. Results of top enriched clusters from Metascape

Supplementary Table 5. CombiROC results (intraindividual and T0 proteomic correlation)

Supplementary Table 6. Metabolites identified in the CSF

Supplementary Table 7. Pathway enrichment among clustered co-regulated metabolites from Fig. 6H

Supplementary Table 8. VSN normalize proteomic data

Supplementary Table 9. Protein differential expression analyses

## Data Availability

All data are available in the main text or the supplementary materials.
