# Supplementary figures and images for: Multi-omics profiling of CSF from spinal muscular atrophy type 3 patients after nusinersen treatment: a 2-year follow-up multicenter retrospective study
Source: Cell Mol Life Sci. 2023 Aug 5;80(8):241. doi: 10.1007/s00018-023-04885-7 (PMC10404194; doi:10.1007/s00018-023-04885-7)

A

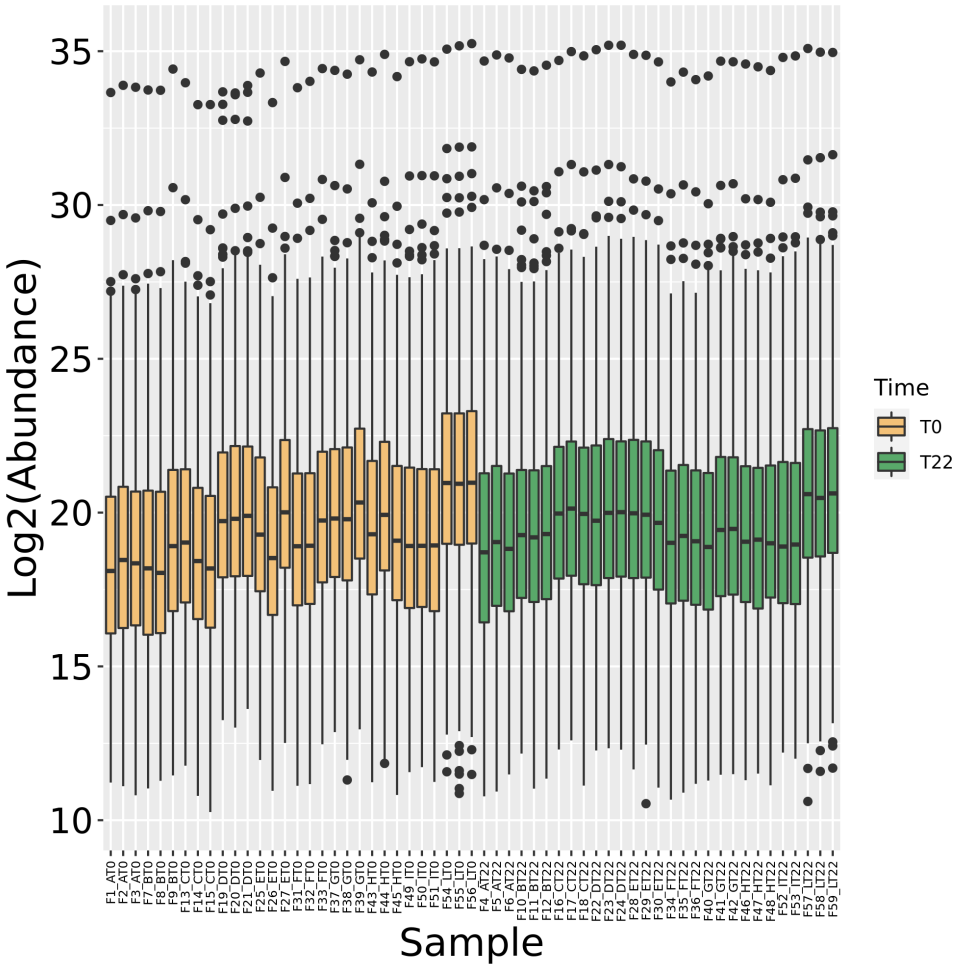

## Suppl. Figure 2

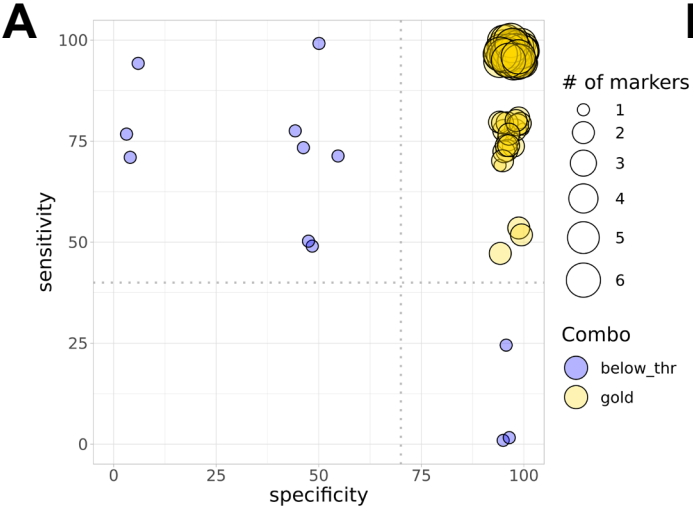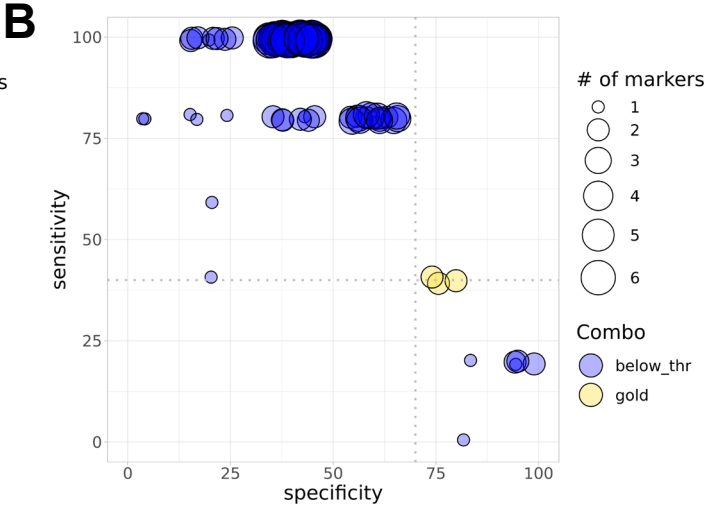

A

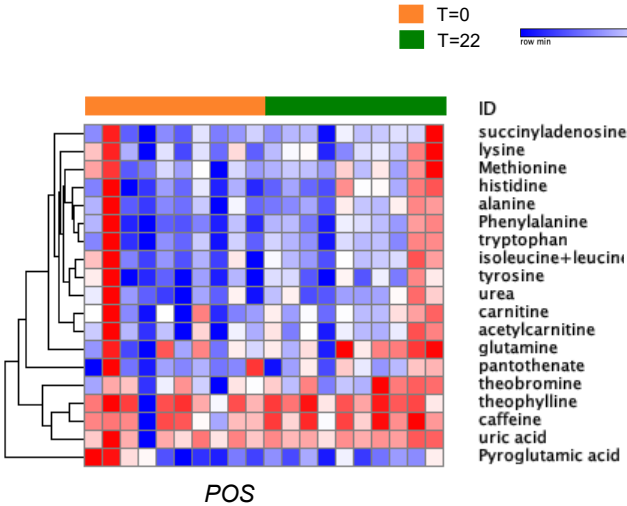

B

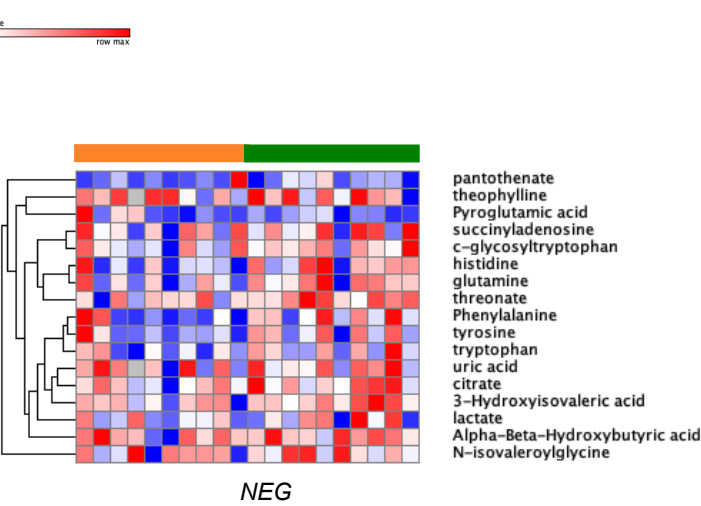

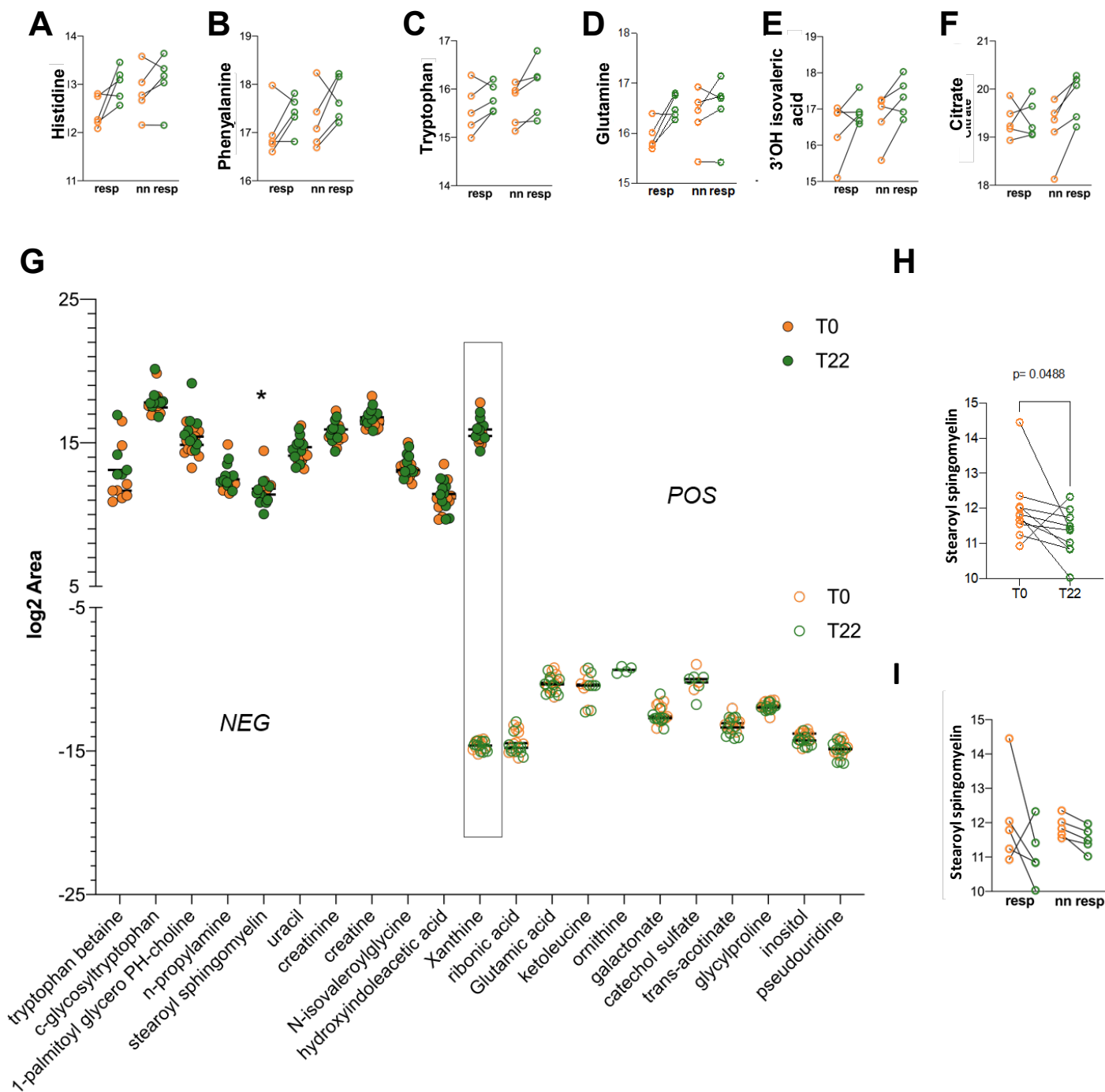

Supplement: Supplementary file 1 — Supplementary Figure 1. Box plot of non-normalized proteomic data, before (yellow, T0) and after treatment (green, T22). Each box plot corresponds to a sample technical replica and describes the distribution of the expression of all proteins detected in each sample. Supplementary Figure 2. A-B. Bubble plots of CombiROC analysis performed testing a combination of up to 6 protein markers among 26 significantly differentially expressed (T22-T0) values in correlation with the classification of responder and non-responder according to RULM and 6MWT clinical score respectively. Blue circles correspond to combinations with specificity <70% and sensitivity <40%, while yellow circles to specificity >70% and sensitivity >40%. Supplementary Figure 3. Hierarchical clustering of normalized detected positive (left panel) and negative (right panel) metabolite level, before (orange bar) and after (green bar) treatment. Supplementary Figure 4. A-F. Paired dot plots for normalized detected positive and negative metabolite levels, before (orange) and after (green) treatment, accounting for HFMSE score (responder and non-responder). G. Dot plot distribution of positive (filled dot) and negative (open dot) metabolites detected with lower confidence, before (orange) and after (green) treatment. In the grey frame metabolites detected in both modes are highlighted. H-I. Paired dot plot for stearoyl sphingomyelin before (orange) and after (green) treatment and accounting for HFMSE score (responder and non-responder) [file 18_2023_4885_MOESM1_ESM.pdf]
